# Curcumin-Loaded Ligand-Conjugated Chitosan Nanoparticles: A Comparative Study of Folic Acid, Phenylalanine, and Butyric Acid Conjugates for Colorectal Cancer

**DOI:** 10.3390/polym18172064

**Published:** 2026-08-25

**Authors:** Chayut Fongsuk, Chutwadee Krisanapun, Duangratana Shuwisitkul

**Affiliations:** 1 Department of Pharmaceutical Technology, Faculty of Pharmacy, Srinakharinwirot University, Ongkharak Campus, Nakhon Nayok 26120, Thailand; chayutmd@gmail.com; 2 Department of Biopharmacy, Faculty of Pharmacy, Srinakharinwirot University, Ongkharak Campus, Nakhon Nayok 26120, Thailand; chatwadee@g.swu.ac.th

**Keywords:** chitosan nanoparticles, curcumin, ligand, colorectal cancer, targeted delivery

## Abstract

Colorectal cancer therapy requires drug delivery systems that improve treatment efficacy and minimize systemic toxicity. In this study, chitosan-based nanoparticles were fabricated and functionalized with folic acid (FA), phenylalanine (PA), and butyric acid (BA) to enhance the delivery of curcumin to Caco-2 cancer cells. The nanoparticles were prepared using an ionic gelation method, and their physicochemical properties, cellular uptake efficiency, and cytotoxicity—including safety evaluation against normal HIEC-6 cells—were investigated. Results showed that ligand conjugation significantly influenced the physicochemical properties of the nanoparticles. CRFANP (FA-modified) exhibited the largest particle size (263.5 nm) due to its rigid aromatic structure, while CRPANP (PA-modified) showed an intermediate size (138.0 nm) and the lowest surface charge (15.59 mV). In contrast, CRBANP (BA-modified) presented the smallest particle size (128.2 nm) and the highest positive surface charge (23.27 mV). These distinct physicochemical properties directly influenced their cellular interactions; CRBANP and CRFANP showed higher uptake than CRPANP, with CRBANP yielding the maximum accumulation of curcumin in Caco-2 (21.92 nM/mg protein) and HT-29 cells (22.09 nM/mg protein). Correlating with the uptake data, cytotoxicity assays revealed that CRBANP was the most potent formulation, exhibiting the lowest IC_50_ of 1.30 µM in Caco-2 cells, which was significantly lower than that of CRFANP (3.49 µM) and CRPANP (7.40 µM), while demonstrating high selectivity against Caco-2 cells with an SI of 46.5 and no apparent toxicity toward normal HIEC-6 cells. This enhanced efficacy is attributed to the synergistic action of butyric acid as a histone deacetylase inhibitor (HDACi), which complements curcumin’s anticancer activity. These findings indicate that integrating butyric acid into chitosan nanoparticles provides an effective and selective strategy for targeted colorectal cancer therapy.

## 1. Introduction

Colorectal cancer (CRC) is a major global oncology issue, with over 1.9 million new cases and approximately 900,000 deaths annually [1]. Conventional chemotherapy lacks specificity, leading to systemic toxicity, rapid clearance, and low bioavailability, which damages healthy tissues and reduces antitumor efficacy [2]. Among various natural products, curcumin, a polyphenolic compound extracted from the rhizome of *Curcuma longa* (turmeric), has shown significant potential in cancer therapy. This compound exhibits diverse pharmacological effects, including anti-inflammatory, anti-proliferative, and pro-apoptotic activities. Its practical application, however, is limited by poor pharmacokinetics. Curcumin exhibits poor aqueous solubility, rapid hepatic metabolism, and low bioavailability, limiting stable systemic concentrations [3]. Nanotechnology-based carriers, such as chitosan nanoparticles, are used to improve curcumin delivery and bioavailability.

Among various nanocarriers, chitosan serves as an alternative for nanoparticle synthesis due to its low toxicity [4]. Ligand functionalization improves targeting selectivity to cancer cells and minimizes damage to healthy tissues. Folic acid, phenylalanine, and butyric acid are utilized for drug targeting. Folic acid binds to the folate receptor-α (FR-α), which is overexpressed in epithelial ovarian, breast, and lung cancers, to facilitate receptor-mediated endocytosis [5]. Phenylalanine targets the L-type amino acid transporter 1 (LAT1), which is upregulated in tumors to support cell growth [6]. Butyric acid targets free fatty acid receptors (FFAR1 and FFAR3) and inhibits histone deacetylases (HDACs) to provide additional antitumor activity [7]. These findings establish the basis for utilizing functionalized chitosan nanoparticles to enhance the cellular entry and therapeutic efficiency of natural compounds such as curcumin.

Previous reports support the targeting potential of these ligand-functionalized carriers. Jin et al. (2016) demonstrated that folate-conjugated chitosan nanoparticles increased ursolic acid uptake 3- to 5-fold in Caco-2 cells compared to unmodified carriers [8]. Following a similar trend in efficacy enhancement, Jatesuda et al. (2016) reported a 3-fold increase in cellular uptake in A549 cells using phenylalanine-modified nanoparticles [9]. This strategy extends to short-chain fatty acids in the work of Park et al. (2020), who observed that butyric acid-functionalized nanoparticles showed a 2-fold higher uptake in Caco-2 cells with a decreased IC_50_ [10]. These functionalized carrier-specific modifications improve drug internalization and antitumor activity.

Several studies have investigated folic acid, phenylalanine, or butyric acid modifications separately. Variations in polymer parameters and testing conditions across those reports prevent direct comparison. This work evaluates FA, PA, and BA modifications under identical experimental conditions. The evaluation isolates ligand effects on cell uptake and cytotoxicity, where the butyric acid formulation exhibits histone deacetylase (HDAC) inhibitory activity to enhance curcumin delivery. This comparative approach identifies the optimal configuration for maximum drug delivery and therapeutic efficacy.

## 2. Materials and Methods

### 2.1. Chemicals

Low molecular weight chitosan (LMWC, 50–190 kDa, 75% deacetylation) and folic acid (FA) were obtained from Tokyo Chemical Industry (TCI, Tokyo, Japan). The viscosity-average molecular weight (*M_v_*) of the starting LMWC was experimentally determined to be in the range of 42,646–53,625 Da (with an average intrinsic viscosity [η] of 4.76 mL/g) using the dilute solution viscosity method. Briefly, chitosan solutions were prepared at five different concentrations ranging from 0.01 to 0.1% *w*/*v* in a solvent system of 0.1 M acetic acid and 0.2 M NaCl. The efflux times were measured using a Ubbelohde viscometer maintained at a constant temperature of 25 °C. The intrinsic viscosity was determined by linear regression of the reduced viscosity (*η*_*sp*_/C, where *η*_*sp*_ is the specific viscosity and C is the concentration) against concentration to zero concentration, and the (*M_v_*) was calculated using the Mark–Houwink equation ($[\eta ]\;=\;KM_{v}^{\alpha }$), where the constants K = 1.81 × 10^−3^ mL/g and α = 0.93 were adopted from established Reference [11] for this solvent-temperature system.

Curcumin (CUR, ≥94% purity), butyric acid (BA, ≥99%), L-phenylalanine (PA, ≥98%), 4-dimethylaminopyridine (DMAP, ≥99%), and N,N′-dicyclohexylcarbodiimide (DCC, 99%) were purchased from Sigma-Aldrich (St. Louis, MO, USA). The 3-(4,5-dimethylthiazol-2-yl)-5-(3-carboxymethoxyphenyl)-2-(4-sulfophenyl)-2H-tetrazolium (MTS) cell proliferation assay kit was purchased from Abcam (Cambridge, UK). All other chemicals and reagents were of analytical grade and used as received without further purification. Ultra-pure water (Milli-Q) was used throughout the experimental procedures.

### 2.2. Preparation of Ligand—Functionalized Chitosan

Folic acid (FA), L-phenylalanine (PA), and butyric acid (BA) were employed as targeting ligands. To initiate the coupling reaction, FA was dissolved in 4 mL of 0.01 N NaOH, whereas PA was dissolved in 4 mL of 0.9% NaCl with heating at 50 °C. For BA, a volume of 4 mL was directly pipetted. Each ligand solution was mixed with 12.22 mg of 4-dimethylaminopyridine (DMAP) as a catalyst. Separately, 41.26 mg of N,N′-dicyclohexylcarbodiimide (DCC) was dissolved in 1 mL of dimethyl sulfoxide (DMSO). The ligand-DMAP and DCC solutions were then combined and stirred at room temperature overnight to activate the carboxyl groups of the ligands.

Simultaneously, chitosan (CS) was solubilized in a 1% *v/v* acetic acid solution, with the pH adjusted to 5.0 using 4 N NaOH. The activated ligand mixture was added dropwise to the chitosan solution under continuous stirring at room temperature for three hours to allow for conjugation. The byproduct, dicyclohexylurea (DCU), appeared as a white precipitate and was removed via filtration. The final conjugated products were then freeze-dried for further use. The specific molar ratios of chitosan to each ligand are summarized in Table 1.

#### 2.2.1. Determination of Chitosan-Ligand Conjugation Efficiency

To determine the conjugation efficiency, 1 mL of the ligand-chitosan solution was centrifuged at 12,000 rpm for 10 min using a centrifuge filter. The filtrate, containing the unconjugated ligands, was then collected and quantified.

The concentrations of folic acid (FA) and phenylalanine (PA) were measured using UV-Vis spectrophotometry at wavelengths of 365 nm and 260 nm, respectively.

For butyric acid (BA), high-performance liquid chromatography (HPLC) was employed with a C18 column (5 μm, 300 mm × 4.6 mm) maintained at 30 °C. The mobile phase consisted of 10 mM KH_2_PO_4_ and acetonitrile (80:20, *v*/*v*) at a flow rate of 1.0 mL/min. Detection was carried out using a UV detector at 210 nm with an injection volume of 10 μL. The conjugation efficiency (CE) was calculated using the following equation:

(1) $$\%\mathrm{CE}\;=\;\left(\frac{\mathrm{Total}\;\mathrm{ligand}\;\mathrm{amount}-\mathrm{Residual}\;\mathrm{ligand}\;\mathrm{amount})}{\mathrm{Total}\;\mathrm{ligand}\;\mathrm{amount}}\;\times \;100\right)$$

#### 2.2.2. Determination of the Degree of Substitution

The degree of substitution (DS) and the percentage of remaining free amine groups of the functionalized chitosan were determined using Equations (2) and (3), respectively:

(2) $$\mathrm{DS}\;=\;\frac{n_{\mathrm{bound}}\;\times \;\%\mathrm{CE}}{n_{\mathrm{glucosamine}}}$$

(3) $$\mathrm{Free}\;\mathrm{amine}\;\left(\%\right)\;=\left(1\;-\mathrm{DS}\right)\times 100$$

where n_bound_ represents the molar amount of the bound ligand, %CE is the conjugation efficiency determination from the unbound ligand analysis, and n_glucosamine_ represents the total molar equivalents of glucosamine units calculated from the mass and degree of deacetylation of chitosan (4.685 × 10^−4^ mol).

### 2.3. Preparation of Blank and Curcumin-Loaded Chitosan Nanoparticles

Blank chitosan nanoparticles were prepared via ionic gelation. Chitosan was dissolved in 20 mL of 1% (*v*/*v*) acetic acid to a final concentration of 1 mg/mL, and the pH was adjusted to 5.0 using 4 N NaOH. Under continuous stirring at 1200 rpm, 1 mL of tripolyphosphate (TPP) solution (10.0 mg/mL in deionized water) was added dropwise at a rate of 0.5 mL/min. The mixture was stirred for an additional 60 min to ensure complete nanoparticle formation.

For curcumin-loaded nanoparticles, a similar procedure was followed. Briefly, 1 mL of curcuminoid solution (1.0 mg/mL in DMSO) was added to the pH-adjusted chitosan solution and stirred for 60 min to allow for drug-polymer interaction. This initial formulation design maintains a fixed 5% (*w*/*w*) curcumin loading ratio relative to the chitosan matrix backbone based on systematic optimization profiles. The TPP solution was then added as previously described. An aliquot of the native nanoparticle suspension was sampled immediately to measure particle size, polydispersity index, and zeta potential via dynamic light scattering. To determine encapsulation efficiency and drug loading capacity, a separate aliquot of the suspension was centrifuged at 12,000 rpm for 60 min to isolate the supernatant containing the unencapsulated drug. The remaining suspension was centrifuged under identical conditions, and the obtained pellet was resuspended in deionized water and freeze-dried to produce the final nanoparticle powder.

### 2.4. Characterization of Ligands Conjugated Chitosan Nanoparticles

#### 2.4.1. Particle Size, Size Distribution, and Zeta Potential

The particle size, polydispersity index (PDI), and zeta potential of the nanoparticles were determined using dynamic light scattering (DLS) and electrophoretic light scattering, respectively, with a Zetasizer (Malvern Instruments, Malvern, UK). Prior to measurement, 1 mL of the nanoparticle suspension was diluted 20-fold with deionized water to ensure optimal signal intensity. All measurements were conducted at a fixed scattering angle of 90° and a controlled temperature of 25 °C.

#### 2.4.2. Nanoparticle Tracking Analysis (NTA)

The particle size distribution and number concentration of curcumin-loaded chitosan nanoparticles, functionalized with folic acid, butyric acid, and phenylalanine, were analyzed using a NanoSight NS300 (Malvern Panalytical, Malvern, UK). This system featured a 405 nm semiconductor blue laser (12 mW) and an EMCCD camera (1920 × 1440 pixels). Samples were introduced into a Nanosight LM10 flow cell (100 µm optical path depth) and analyzed using NTA software version 3.2. The detection threshold was set at 4 for all measurements. Measurements were performed at an instrument-recorded temperature of 25.0 °C utilizing a 488 nm laser wavelength and a fixed scattering angle of 90°. The software tracked the Brownian motion of individual particles to determine their hydrodynamic diameters and particle number concentrations. Each formulation was measured in five replicates, with results reported as mode diameter and percentile diameters (D_10_, D_50_ and D_90_).

#### 2.4.3. Entrapment Efficiency (EE) and Curcumin Loading Capacity (CLC)

The amount of curcumin encapsulated within the nanoparticles was determined indirectly by measuring the unentrapped drug. The nanoparticle suspension was centrifuged at 10,000 rpm for 20 min to separate the particles from the supernatant. The supernatant was then carefully collected, and the washings were combined with the supernatant for analysis. The concentration of curcumin in the supernatant was quantified using a UV-Vis spectrophotometer (Shimadzu, Kyoto, Japan) at 425 nm against a proper blank solution. The EE and LE were calculated using the following equations:

(4) $$\mathrm{EE}\;=\;\left(\frac{\mathrm{Total}\;\mathrm{amout}\;\mathrm{of}\;\mathrm{curcumin}\;\;-\;\mathrm{Free}\;\mathrm{curcumin}}{\mathrm{Total}\;\mathrm{amount}\;\mathrm{of}\;\mathrm{curcumin}}\right)\times 100$$

(5) $$\mathrm{CLC}=\left(\frac{\mathrm{Total}\;\mathrm{amout}\;\mathrm{of}\;\mathrm{curcumin}\;-\mathrm{Free}\;\mathrm{curcumin}}{\mathrm{Total}\;\mathrm{amount}\;\mathrm{of}\;\mathrm{Nanoparticles}}\right)\;\times \;100$$

All measurements were performed in triplicate, and the results are expressed as mean ± standard deviation (SD).

#### 2.4.4. Fourier Transform Infrared (FTIR) Spectroscopy

To confirm the successful conjugation of ligands to the chitosan backbone and the encapsulation of curcumin, FTIR analysis was performed. Samples of pure chitosan, pure ligands (FA, PA, and BA), and the functionalized nanoparticles were analyzed using an Attenuated Total Reflection (ATR) accessory. The spectra were recorded using an FTIR spectrophotometer (Shimadzu, Japan) over a scanning range of 4000 to 400 cm^−1^ with a resolution of 4 cm^−1^. The formation of amide linkages was identified by monitoring the characteristic shifts in the carbonyl (C=O) and amine (N-H) stretching frequencies.

### 2.5. Cytotoxicity Studies

The Caco-2 (HTB-37), HT-29 (HTB-38), and HIEC-6 (CRL-3266) cell lines were purchased from the American Type Culture Collection (ATCC, Manassas, VA, USA). The cytotoxicity of the curcumin solution (CR) and various nanoparticle formulations (CR-CSNPs, CR-CFANPs, CR-CBANPs, and CR-CPANPs) was evaluated against two human colon carcinoma cell lines (Caco-2 and HT-29) and a normal human intestinal epithelial cell line (HIEC-6) using the MTS assay.

Cells were maintained in their specific growth media—Minimum Essential Medium (MEM) for Caco-2, McCoy’s 5A medium for HT-29, and Opti-MEM for HIEC-6—each supplemented with 10% fetal bovine serum (FBS), and incubated at 37 °C in a humidified atmosphere with 5% CO2.

For the assay, cells were seeded into 96-well plates at a density of 1 × 10^4^ cells/well and incubated for 24 h to allow for attachment. The cells were then treated with the drug or nanoparticle formulations at various concentrations and incubated for an additional 24 h. Cells incubated without treatment served as the control group. The final concentration of DMSO in all experimental groups was maintained at 1% (*v*/*v*). Preliminary screening confirmed that this DMSO concentration showed no cytotoxic effect on Caco-2, HT-29, or HIEC-6 cells, confirming that the observed antitumor activities were related to the drug-loaded nanoparticle systems. After the incubation period, MTS solution was added to each well, and the plates were incubated for a further 4 h. The cell viability was quantified by measuring the absorbance at 490 nm using a microplate reader (LUMIstar Omega, Ortenberg, Germany). The results were used to determine the half-maximal inhibitory concentration (IC_50_) for each formulation. The selectivity index (SI) was calculated to assess the safety of the nanoparticles toward normal cells compared to cancerous cells.

### 2.6. Measurement of Cellular Uptake

To evaluate the internalization of curcumin, both quantitative analysis via HPLC and qualitative imaging via fluorescence microscopy were performed.

#### 2.6.1. Quantitative Cellular Uptake by HPLC

Caco-2 and HT-29 cells were seeded at 1 × 10^5^ cells/well in 24-well plates and incubated for 24 h. After washing with PBS, cells were treated for 2 h with either free curcumin (50 µM in 0.5% DMSO) or various nanoparticle formulations (CR-CSNPs, CR-CFANPs, CR-CBANPs, and CR-CPANPs) equivalent to 50 µM curcumin.

Post-incubation, cells were washed, harvested with trypsin-EDTA, and lysed in 1000 µL of 70% methanol using probe sonication (3 cycles, 20 s on/10 s off). The lysate was centrifuged (15,000 rpm for 10 min), filtered (0.2 µm), and analyzed by HPLC.

HPLC conditions: An Inertsil ODS-3 column (4.6 × 300mm, 5 µm) was used at 30 °C. The mobile phase was acetonitrile/2% *v/v* acetic acid in water (50:50, *v*/*v*), delivered at 1.5 mL/min. Detection was at 425 nm with a 20 µL injection volume.

Quantification: Curcumin concentration was determined using a standard calibration curve (0.1–1.0 µg/mL, R2 > 0.999). Uptake was normalized to total cellular protein content, measured using the Bio-Rad Bradford assay with BSA as the standard (100–500 µg/mL range, measured at 595 nm).

#### 2.6.2. Intracellular Localization by Fluorescence Microscopy

Qualitative curcumin uptake was assessed via confocal microscopy. Caco-2 cells were seeded in 12-well plates (5 × 10^4^ cells/mL) and incubated for 24 h. Cells were treated with 20 µM of free curcumin or nanoparticle formulations for 4 h.

Post-treatment, cells were washed 3 times with cold PBS and fixed using 4% paraformaldehyde for 20 min. After washing again, the intrinsic fluorescence of internalized curcumin was immediately observed and imaged using a confocal microscope.

### 2.7. Statistical Analysis

All quantitative data were expressed as mean ± standard deviation (SD). Statistical differences between groups were determined using one-way analysis of variance (ANOVA) followed by Tukey’s post hoc test. Differences were considered statistically significant when the *p*-value was less than 0.05 (*p* < 0.05). All analyses were performed using software such as GraphPad Prism (Version 9.0) or SPSS (Version 21.0).

## 3. Results

The conjugation of ligands to chitosan (CS) was performed using carbodiimide chemistry with dicyclohexylcarbodiimide (DCC) as the coupling agent to form amide linkages without spacer molecules. DCC activates the carboxylic groups of ligands, including folic acid (FA), butyric acid (BA), and phenylalanine (PA), generating reactive intermediates for subsequent bonding with the amino groups of chitosan. To ensure high chemical purity, a two-step synthesis method was used instead of a one-step approach to prevent byproduct contamination. In a one-step method, simultaneous mixing generates the insoluble dicyclohexylurea (DCU) byproduct, which is difficult to filter from the polymer solution. Pre-activating the ligand carboxyl groups in an isolated organic phase enables the solid DCU to be filtered out completely before combining with the chitosan matrix. This system avoids retaining the byproduct in the final reaction mixture and evaluates ligand conjugation efficiency prior to nanoparticle fabrication.

### 3.1. Characterization of Ligands Conjugated Chitosan

#### 3.1.1. FTIR Analysis

The FTIR analysis effectively confirms the successful conjugation of folic acid, butyric acid and phenylalanine to chitosan. The appearance of new peaks corresponding to amide bonds, along with the disappearance of carboxylic peaks from the ligands, provides strong evidence of chemical linkage. Shifts in characteristic vibrations and the appearance of peaks associated with specific functional groups of each ligand further support the successful chemical modification of the chitosan molecules.

The FTIR spectrum of chitosan (CS) shows characteristic peaks indicative of its functional groups. A broad peak at 3351–3377 cm^−1^ corresponds to O-H and N-H stretching vibrations, while peaks at 2876–2939 cm^−1^ represent C-H stretching. The amide I band, attributed to C=O stretching, is observed at 1648–1655 cm^−1^, and the amide II band, corresponding to N-H bending, appears at 1581–1588 cm^−1^. Additional peaks at 1026–1032 cm^−1^ are attributed to C-O-C stretching in the chitosan backbone.

**Figure 1:**
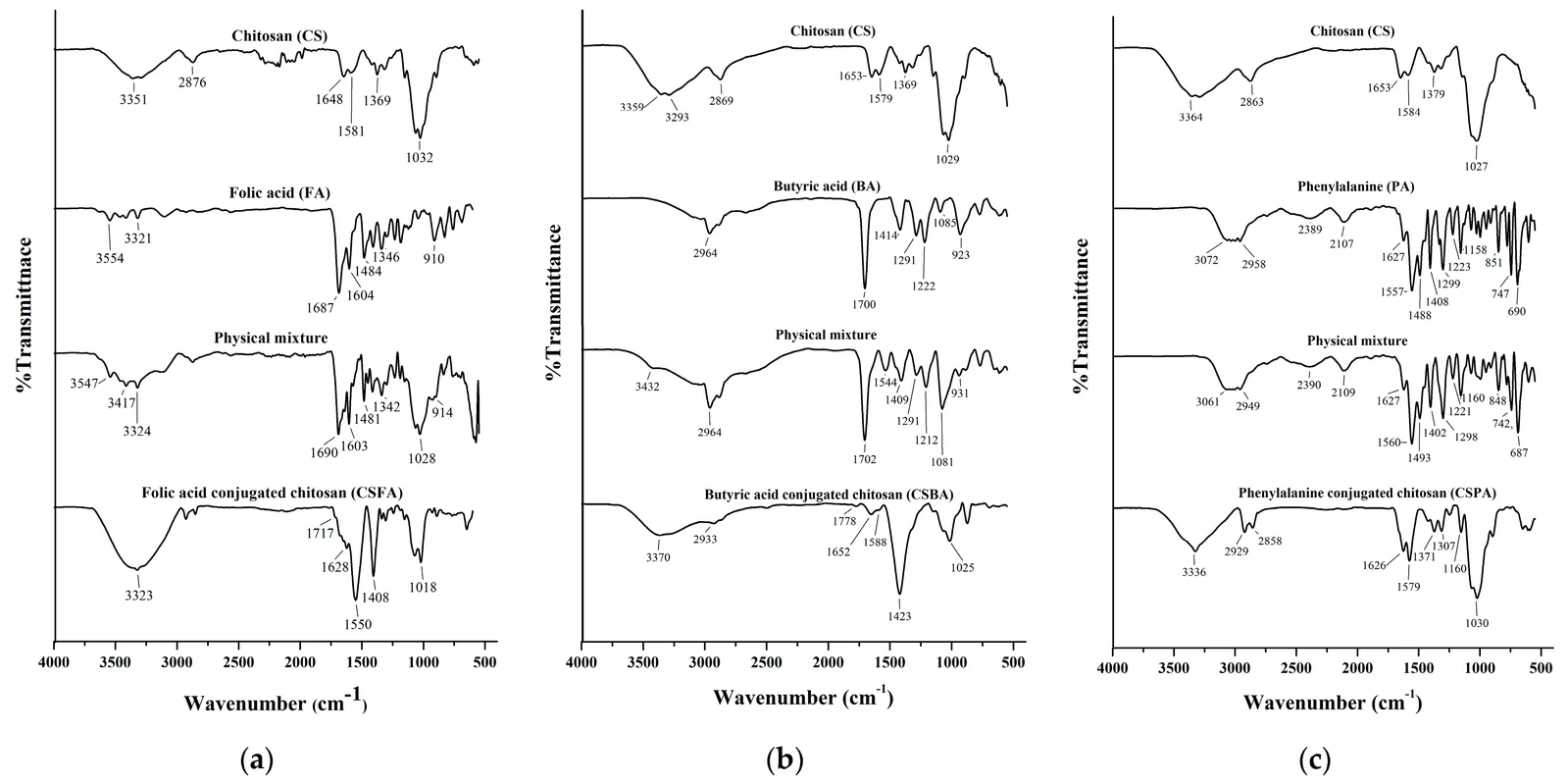
FTIR spectra of chitosan nanoparticles conjugated with various ligands: (**a**) folic acid-conjugated chitosan (CSFA), (**b**) butyric acid-conjugated chitosan (CSBA), and (**c**) phenylalanine-conjugated chitosan (CSPA).

The FTIR spectrum of folic acid (FA) displays a sharp C=O stretching peak at 1687 cm^−1^, characteristic of its carboxylic groups, and multiple peaks between 1400 and 1600 cm^−1^ corresponding to aromatic C=C vibrations. As shown in the FTIR spectrum of CSFA in Figure 1a, a new peak at 1717 cm^−1^ indicates the formation of an amide bond between the carboxylic group of FA and the amino group of CS. The peak at 1550 cm^−1^ (N-H bending) and the broadening at 3323 cm^−1^ (O-H and N-H stretching) further support successful conjugation. The disappearance of the C=O peak at 1687 cm^−1^ in pure FA confirms the reaction.

The FTIR spectrum of butyric acid (BA) exhibits a sharp peak at 1700 cm^−1^, which corresponds to C=O stretching from its carboxylic group, along with CH stretching vibrations in the range of 2964–2883 cm^−1^. In the CSBA spectrum represented in Figure 1b, the disappearance of the C=O peak at 1700 cm^−1^ and the appearance of a new peak at 1652 cm^−1^ confirm the formation of an amide bond [12,13]. Additionally, peaks at 2935 and 2865 cm^−1^, attributed to CH stretching of the hydrocarbon chains, further indicate the successful incorporation of BA into the chitosan structure.

The FTIR spectrum of phenylalanine (PA) exhibits aromatic C=C stretching peaks near 1500–1600 cm^−1^ and a peak at 1704 cm^−1^ corresponding to the carboxylic group. The CSPA spectrum in Figure 1c reveals the disappearance of the carboxylic peak at 1704 cm^−1^ and the appearance of a new peak at 1655 cm^−1^ confirm the formation of an amide bond. Peaks at 1579 cm^−1^ (N-H bending) and 3336 cm^−1^ (N-H stretching) further indicate successful conjugation [4]. Shifts in aromatic vibrations also validate the attachment of PA to CS.

#### 3.1.2. Conjugation Efficiency

Ligand selection and initial concentrations significantly influenced the chemical modification of the chitosan (CS) backbone. The optimal concentration for achieving maximum conjugation efficiency varied across the different ligands. For CS:FA, the efficiency peaked at a 1:200 ratio (88.87 ± 0.24%) before decreasing at higher concentrations. In contrast, the efficiency for CS:BA increased progressively with higher initial concentrations, reaching its highest value of 84.43 ± 0.20% at a 1:300 ratio. Conversely, CS:PA showed a sharp reduction in efficiency as the ligand concentration exceeded 1:50, dropping significantly to 24.33 ± 0.65% at the 1:300 ratio.

Despite these variations in efficiency percentages, the actual bound ligand (mol/mol) increased within each group in response to higher initial amounts, leading to a steady decrease in available amine groups on the chitosan chain, as detailed in Table 2. At the 1:300 ratio, the available free amine groups were below the detection threshold. This reduced detection relates to steric hindrance from the dense ligand packing on the polymer surface, which restricts chemical reagent access during analysis. FTIR analysis confirms that the structural integrity of the functionalized chitosan is maintained without polymer degradation, indicating that the covalent amide linkage remains the primary structural modification.

For curcumin-loaded nanoparticles prepared via ionic gelation, the 1:100 ratio provided the most balanced physicochemical properties for all three ligands. This specific setting maintained available amine groups between 58.20% and 63.24%, ensuring sufficient cationic sites for effective ionic crosslinking with tripolyphosphate (TPP). Such a balance is necessary to achieve colloidal stability and a desirable positive zeta potential. At this stage, the actual bound ligands (70.93 to 244.25 mol/mol) introduce enough hydrophobicity to improve curcumin encapsulation through hydrophobic interactions and π–π stacking while preserving the physical integrity of the resulting particles.

### 3.2. Characterization of Chitosan Nanoparticles

#### 3.2.1. Size, Size Distribution and Zeta Potential

The particle size, polydispersity index (PDI), and zeta potential of the ligand-functionalized chitosan nanoparticles, prepared at a CS:ligand molar ratio of 1:100, were investigated using a DLS instrument. The results for formulations conjugated with folic acid, butyric acid, and phenylalanine are presented in Table 3.

The average particle size varied significantly among the three formulations. CRFANPs exhibited the largest particle size at 263.5 ± 8.4 nm with a PDI of 0.174 ± 0.037, indicating a relatively narrow and uniform size distribution. In contrast, CRBANPs had the smallest particle size of 128.2 ± 3.0 nm but displayed a broader size distribution, as evidenced by a higher PDI of 0.349 ± 0.005. Similarly, CRPANPs showed an intermediate particle size of 138.0 ± 2.9 nm with a PDI of 0.340 ± 0.028. The significantly larger size of CRFANPs suggests that the structural characteristics of the folic acid molecule play a decisive role in nanoparticle matrix formation, primarily through steric hindrance that restricts tight molecular packing. Furthermore, the higher PDI values for CRBANPs and CRPANPs reflect a less uniform distribution, which is likely associated with variations in surface modification patterns or inter-particle interactions during the ionic gelation process.

The zeta potential, which reflects surface charge and colloidal stability, also varied among the formulations despite the consistent molar ratio used. CRBANPs exhibited the highest zeta potential at 23.27 ± 1.10 mV, indicating a strong positive charge that supports colloidal stability and effective cellular interaction. CRFANPs showed a moderately positive zeta potential of 17.05 ± 0.74 mV, while CRPANPs had the lowest value at 15.59 ± 2.14 mV. Although these values were lower than those of CRBANPs, they remain within a range sufficient to provide adequate electrostatic stability for biological applications. These findings indicate that the chemical structure of the conjugated ligand significantly influences the surface charge density of the nanoparticles, with the aliphatic butyric acid modification better preserving the chitosan’s cationic properties compared to the aromatic ligand modifications.

#### 3.2.2. Nanoparticle Tracking Analysis (NTA): Size Distribution and Concentration

The average particle size of the ligand-conjugated nanoparticles varied significantly among the three formulations. According to the results in Table 4, CRFANP exhibited the largest dimensions at 263.5 ± 8.4 nm with a PDI of 0.174 ± 0.037, indicating a relatively narrow and uniform distribution. In contrast, CRBANP possessed the smallest diameter (128.2 ± 3.0 nm) but showed a broader size range, as evidenced by a higher PDI of 0.349 ± 0.005. Similarly, CRPANP displayed an intermediate size of 138.0 ± 2.9 nm with a PDI of 0.340 ± 0.028, reflecting a distribution profile comparable to that of CRBANP. The size distribution profiles obtained from NTA are consistent with the trends observed in the Zetasizer measurements, further validating the influence of ligand functionalization on nanoparticle size.

**Figure 2:**
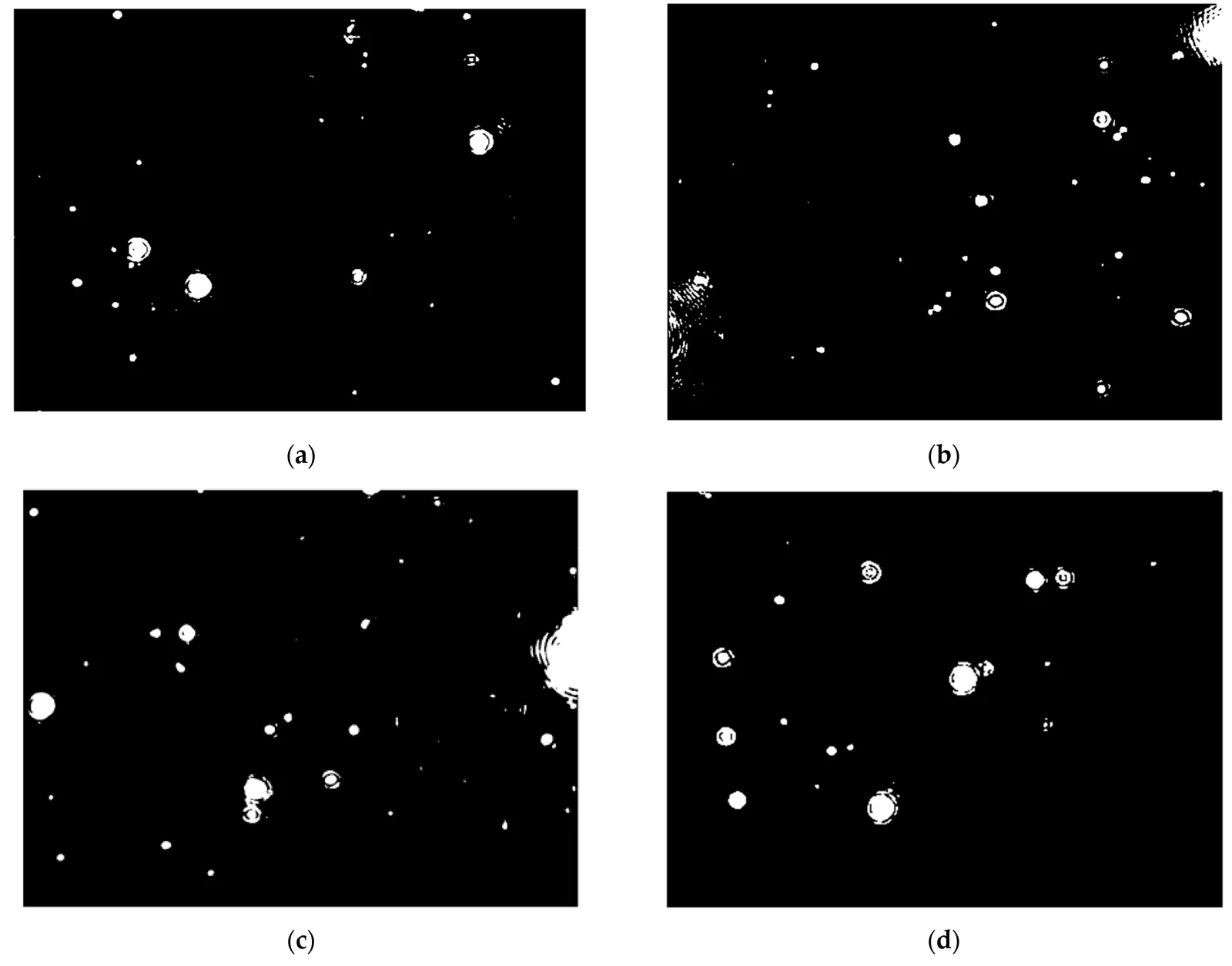
NTA scattering images showing the light scattering profiles of curcumin-loaded chitosan nanoparticles functionalized with various ligands: (**a**) unmodified chitosan (CRCSNP), (**b**) folic acid (CRFANP), (**c**) butyric acid (CRBANP), and (**d**) phenylalanine (CRPANP).

The NTA size distribution profiles of all four formulations are illustrated in Figure 2. Each formulation displayed a broad, right-skewed unimodal distribution typical of polydisperse polymeric nanoparticles. Data presented in Table 4 show that mode diameters—representing the most frequent particle size—were 127.5, 107.5, 232.5, and 107.5 nm for CRCSNP, CRFANP, CRBANP, and CRPANP, respectively. These values confirm that the majority of particles remained well below 250 nm in three of the four groups. The D_10_–D_90_ values detailed in Table 4 were wide across all formulations, ranging from 78 to 429 nm for CRCSNP to 98–567 nm for CRBANP. This indicates a minor fraction of larger aggregates at the upper tail of each distribution, which is reflected in the extended shoulders of the NTA profiles shown in Figure 2. Among the ligand-conjugated samples, CRBANP displayed a distinct distribution profile with a D_50_ of 255 nm. This result corresponds to the NTA mean and DLS Z-average values, reflecting the structural influence of butyric acid conjugation. The inherent flexibility and short aliphatic chain of butyric acid facilitate more dynamic surface properties or specific polymer–ligand arrangements, which contrasts with its distribution characteristics from the more rigid aromatic systems of folic acid and phenylalanine. Such a profile demonstrates the complex interaction between the flexible ligand and the chitosan matrix, which contributes to the enhanced biological efficacy and high positive surface charge observed in the CRBANP formulation.

#### 3.2.3. Encapsulation Efficiency and Drug-Loading Efficiency

The study investigated the effect of conjugating chitosan with different ligands—folic acid, butyric acid, and phenylalanine—at a CS:ligand molar ratio of 1:100—on the entrapment efficiency (EE) and Curcumin Loading capacity (CLC) of nanoparticles as shown in Table 3. The entrapment efficiency (EE) and Curcumin Loading capacity (CLC) of CRCSNPs were 78.64 ± 1.95% and 4.92 ± 0.55%, respectively. Folic acid-conjugated nanoparticles (CRFANP) showed a slight improvement with an EE of 79.84% and an LE of 5.53%. Butyric acid-conjugated nanoparticles (CRBANP) displayed a comparable EE of 77.24% but a slightly lower LE of 4.89%. Phenylalanine-conjugated nanoparticles (CRPANPs) demonstrated an EE of 79.77% and an LE of 4.87%, indicating consistent performance across the functionalized formulations.

The differences in EE and LE can be attributed to the distinct chemical properties of each ligand. Folic acid, with its multiple carboxyl groups, provides more reactive sites for conjugation with chitosan, enhancing both EE and LE. Butyric acid, being a smaller molecule, can efficiently interact with chitosan, resulting in high EE but slightly lower LE due to its lower overall mass contributing to the nanoparticle. Phenylalanine, with its moderate size and reactivity, offers a balanced improvement in both EE and LE, suggesting its suitable incorporation without significant alterations to nanoparticle structure.

### 3.3. Release Profile

**Figure 3:**
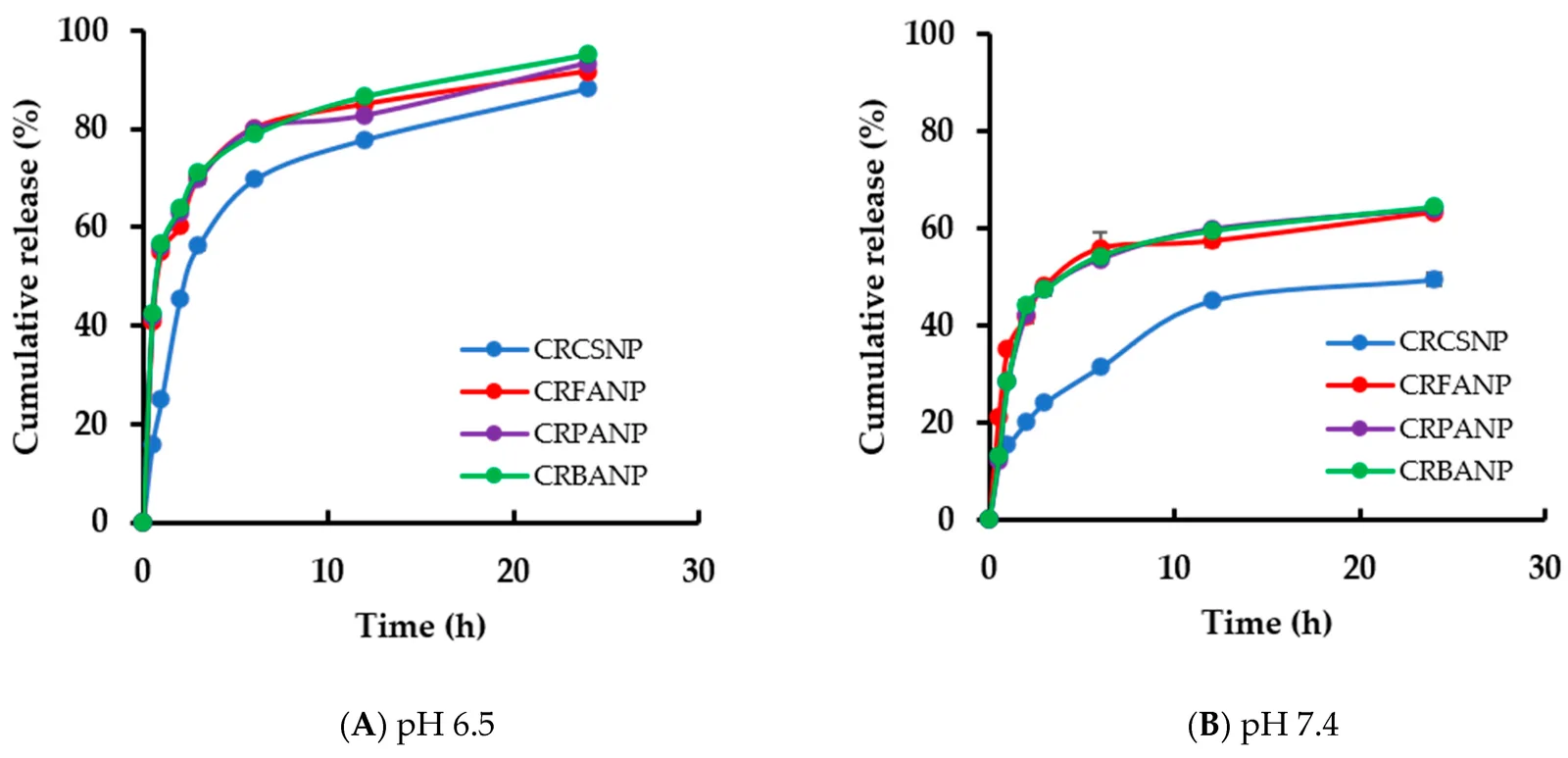
Release profile of nanoparticulate formulations at different pH. Statistical analysis indicates significant differences (*p* < 0.05) between CRCSNP and all functionalized formulations (CRFANP, CRPANP, CRBANP) from 0.5 to 24 h at pH 6.5, and from 1 to 24 h at pH 7.4.

The in vitro release profiles of curcumin from unmodified (CRCSNP) and ligand-functionalized chitosan nanoparticles (CRFANP, CRPANP, and CRBANP) were systematically evaluated under two distinct biomimetic environments: pH 6.5, which replicates the acidic extracellular tumor microenvironment and late endosomes, and pH 7.4, which represents normal systemic physiological conditions. The cumulative percentage of drug release plotted against a 24 h time course provides crucial insights into the structural stability and structural unpacking behavior of the developed polymeric matrices, as illustrated in Figure 3.

At pH 6.5, all formulations exhibited a biphasic release profile with an initial burst phase, followed by a sustained release phase up to 24 h (Figure 3A). Within the first 0.5 h of incubation, the ligand-conjugated formulations (CRFANP, CRPANP, and CRBANP) displayed rapid initial drug dissolution, reaching release values of 40.98 ± 0.08%, 41.79 ± 0.46%, and 42.41 ± 0.14%, respectively, whereas the unmodified CRCSNP exhibited a significantly lower release of 15.81 ± 0.41%. At the 24 h interval, CRBANP yielded the maximum cumulative drug release at 95.17 ± 0.37%, while CRPANP, CRFANP, and CRCSNP reached final cumulative release percentages of 93.31 ± 0.14%, 91.73 ± 0.04%, and 88.28 ± 0.79%, respectively.

A suppressed and controlled drug release pattern was observed for all tested formulations at physiological pH 7.4 (Figure 3B). The initial drug leakage at 0.5 h was restricted to 13.18% ± 0.23% for CRCSNP and ranged between 12.17% ± 0.17% and 21.02% ± 0.02% for the ligand-functionalized nanoparticles. At the 24 h termination point, the total cumulative drug release values plateaued at approximately 63% to 64% for the modified groups, whereas CRCSNP released only 49.46% ± 1.34% of the encapsulated drug. This difference between the two pH environments demonstrates a pH-responsive release characteristic, indicating that the polymeric matrix minimizes premature drug leakage during systemic circulation while triggering accelerated drug release upon entering the acidic tumor microenvironment.

### 3.4. Cytotoxicity

**Figure 4:**
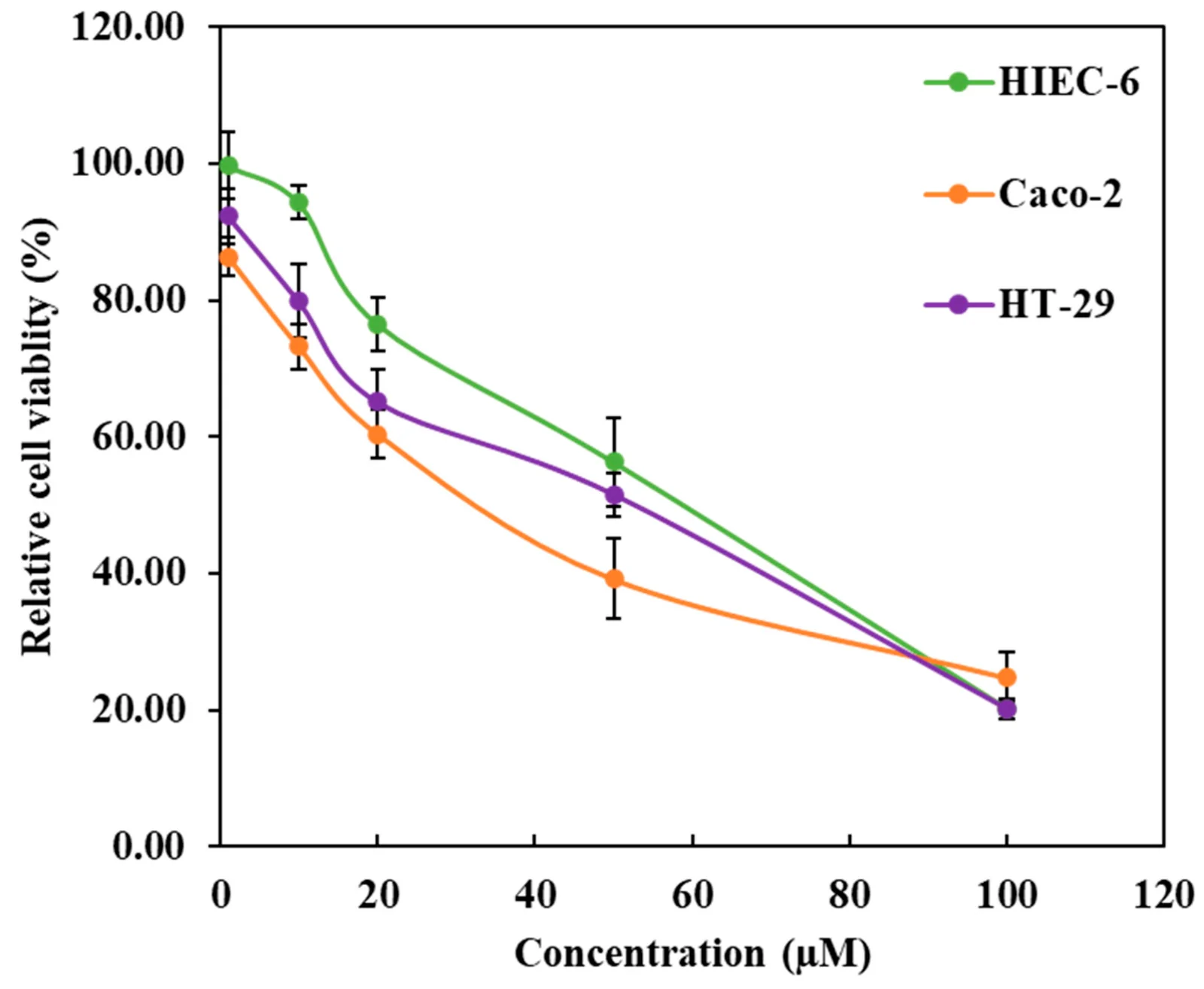
Dose-dependent cytotoxicity of free curcumin (CR) against HIEC-6, Caco-2, and HT-29 cell lines after 24 h of incubation.

The cytotoxicity of free curcumin (CR) and various nanoparticle formulations was evaluated against Caco-2, HT-29, and HIEC-6 cell lines using the MTS assay. The dose-dependent cytotoxicity of free curcumin across all cell lines is presented in Figure 4, while the half-maximal inhibitory concentration (IC_50_) values for all formulations are summarized in Table 5.

All nanoparticle formulations exhibited significantly lower IC_50_ values compared to free curcumin (CR) (*p* < 0.05), indicating enhanced anticancer activity. For Caco-2 cells, free CR had an IC_50_ of 20.97 ± 4.12 µM, which decreased to 7.54 ± 1.25 µM for CRCSNP. Among the ligand-conjugated nanoparticles, CRBANP (butyric acid-conjugated) showed the strongest effect with the lowest IC_50_ of 1.30 ± 0.43 µM, a 16.12-fold increase in potency over free CR, attributed to its dual-action as an HDAC inhibitor. CRFANP also showed high efficacy (3.49 ± 0.73 µM) via folate receptor targeting.

HT-29 cells displayed higher resistance to treatment, but CRBANP and CRFANP were still most effective (IC_50_ around 11–12 µM). CRPANP (phenylalanine-conjugated) was the least effective in both cell lines.

Regarding the safety profile, all nanoparticle formulations exhibited significantly lower toxicity toward normal HIEC-6 cells compared to the cancerous lines. While free CR showed an IC_50_ of 60.45 ± 2.84 µM against HIEC-6, the IC_50_ values for all nanoparticle groups remained not detected (ND) within the tested concentration range. These findings confirm the high selectivity of the developed systems, particularly for CRBANP, which shows a wide therapeutic window by effectively targeting malignant cells while sparing healthy HIEC-6 cells.

All formulations showed significantly higher cell viability in the normal HIEC-6 cells, confirming selective action against cancer cells. The Selectivity Index (SI) was highest for CRBANP against Caco-2 cells (SI = 46.5), representing a high degree of target specificity.

### 3.5. Cellular Uptake Analysis

The cellular internalization of curcumin was investigated using both quantitative HPLC analysis and qualitative confocal laser scanning microscopy (CLSM) to evaluate the targeting efficiency of the functionalized nanoparticles. The results demonstrate the mechanisms through which ligand conjugation enhances drug delivery into colorectal cancer cells.

#### 3.5.1. Quantitative Analysis by HPLC

**Figure 5:**
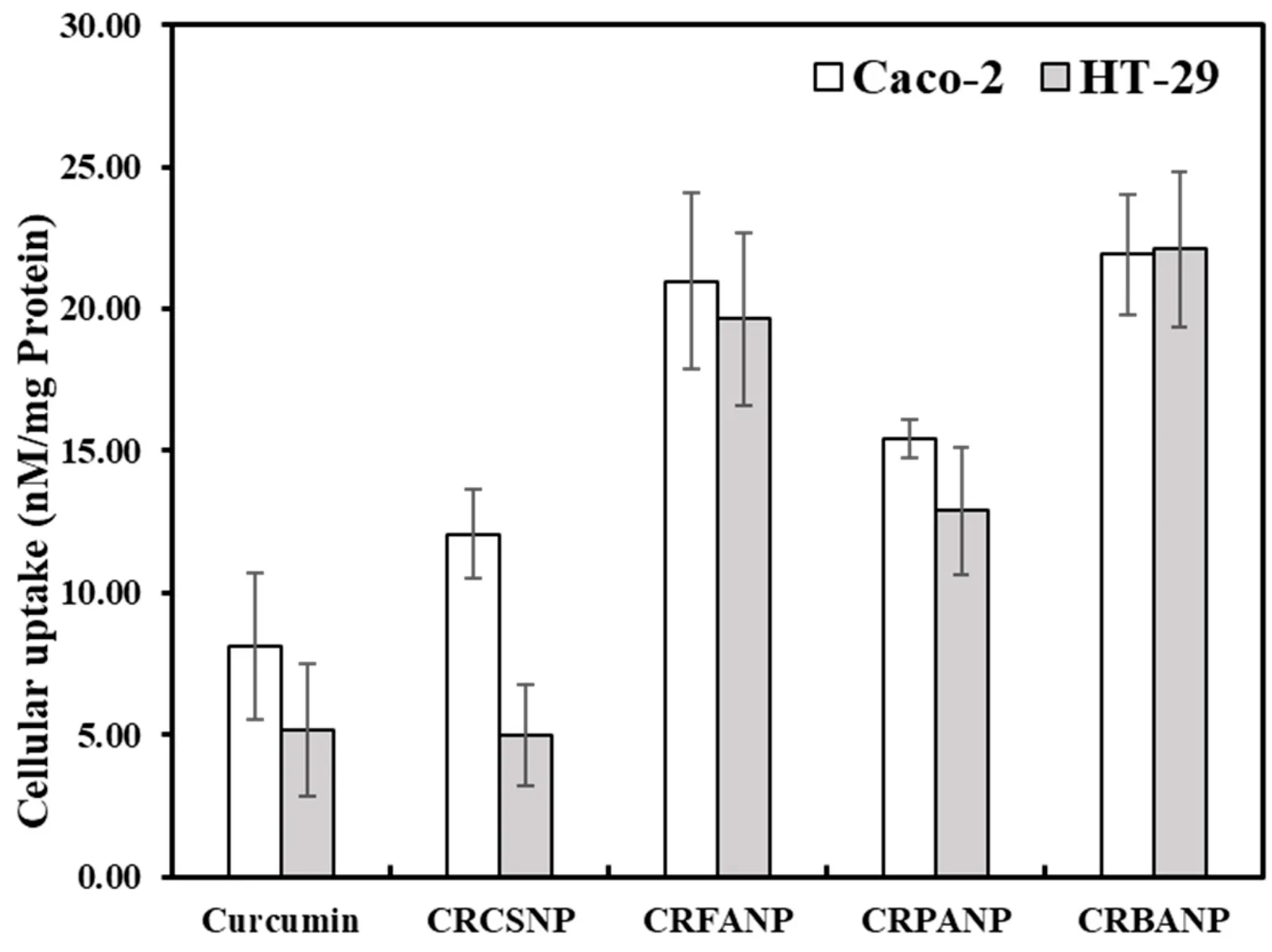
Quantitative cellular uptake of curcumin (nM/mg protein) by Caco-2 and HT-29 cells after 2 h of incubation with various formulations.

To quantify cellular internalization, curcumin was extracted from the cell lysates using 70% methanol with sonication. The extraction efficiency during this process remains equivalent between the free and encapsulated formulations. The 70% methanol solvent induces structural swelling and expands the polymer network by disrupting the intermolecular hydrogen bonding of chitosan [14]. Mechanical sonication further drives matrix fragmentation [15] while prompting protonation of the polymer backbone. These synergistic physical and chemical actions efficiently break down non-covalent interactions and electrostatic bonds between the entrapped drug and the polymer backbone, leading to the complete dissolution of the encapsulated curcumin. The quantitative uptake of curcumin by Caco-2 and HT-29 cells after 2 h of incubation is summarized in Table 6 and Figure 5. Free curcumin (CR) showed the lowest cellular uptake in both cell lines (Caco-2: 8.11 ± 2.59 nM/mg protein; HT-29: 5.16 ± 2.59 nM/mg protein), primarily attributed to its poor aqueous solubility and limited passive diffusion across the lipophilic cell membrane.

Encapsulation within non-functionalized chitosan nanoparticles (CRCSNPs) significantly enhanced uptake (Caco-2: 12.06 ± 1.59 nM/mg protein), representing a 1.5-fold increase. This improvement results from the mucoadhesive properties of chitosan and the electrostatic interactions between the positively charged amino groups of the nanoparticles and the negatively charged components of the cell membrane, facilitating better cellular association.

Ligand-functionalized nanoparticles demonstrated superior internalization efficiency compared to both free CR and CRCSNPs. In Caco-2 cells, CRBANP (butyric acid-conjugated) and CRFANP (folic acid-conjugated) exhibited the highest uptake, measuring 21.92 ± 1.78 and 20.97 ± 3.12 nM/mg protein, respectively. This represents a substantial 2.6–2.7-fold increase compared to free CR. CRPANP (phenylalanine-conjugated) showed a moderate uptake of 15.40 ± 0.68 nM/mg protein. Similar trends were observed in HT-29 cells, with CRBANP showing the highest uptake (22.09 ± 2.73 nM/mg protein).

The high accumulation of CRFANPs is attributed to folate receptor-mediated endocytosis, a pathway highly efficient in cancer cells due to receptor overexpression. The high uptake of CRBANPs, meanwhile, is facilitated by its interaction with short-chain fatty acid (SCFA) receptors and its ability to act as a permeation enhancer, potentially modulating tight junctions to increase drug delivery. This high intracellular accumulation directly correlates with the enhanced cytotoxicity observed for these specific formulations in the IC_50_ study.

#### 3.5.2. Qualitative Analysis by Fluorescence Microscopy

**Figure 6:**
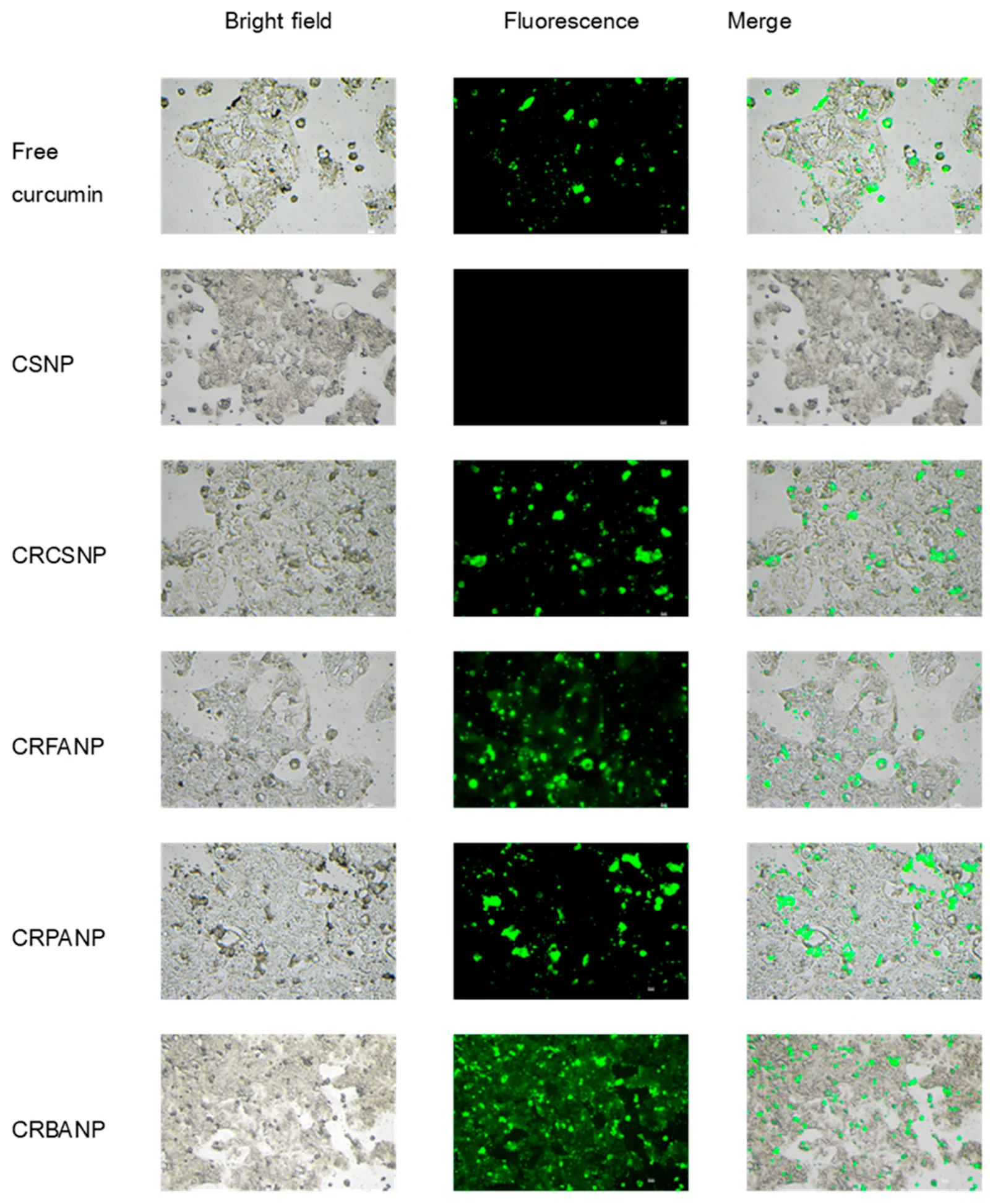
Confocal microscopy images illustrating the internalization of free curcumin and curcumin encapsulated in various nanoparticle formulations by Caco-2 cells after 4 h of incubation. (20× magnification; scale bar = 20 µm).

The enhanced cellular internalization mechanisms were visually confirmed using CLSM, utilizing the intrinsic green fluorescence of curcumin. As shown in Figure 6, cells treated with free curcumin displayed only weak, diffuse fluorescence signals primarily concentrated at the cell periphery, indicating minimal passive diffusion into the intracellular compartment.

In contrast, cells treated with CRCSNP exhibited noticeably stronger green fluorescence signals distributed throughout the cytoplasm, confirming successful internalization via non-specific electrostatic interactions and endocytosis. The most intense and dense fluorescence signals were observed in cells treated with the ligand-functionalized nanoparticles, particularly CRFANP and CRBANP, consistent with the quantitative HPLC data. These images provide direct visual evidence that specific ligand conjugation significantly bolsters the delivery and accumulation of curcumin within the target cancer cells.

## 4. Discussion

Ligand structural features influenced the physicochemical properties of the functionalized nanoparticles, cellular internalization efficiency, and overall therapeutic efficacy of the functionalized chitosan nanoparticles, and the relationships between these factors are evaluated below. FA, PA, and BA modifications are compared directly using identical polymer parameters. Previous studies evaluated these ligands under varying experimental conditions, making direct comparison of their delivery efficiency difficult. The data confirms that butyric acid modification provides potent cytotoxicity to enhance the therapeutic response of the released curcumin. The correlation between these verified structural profiles and the subsequent physicochemical properties is evaluated systematically in the following sections.

### 4.1. Synthesis and Characterization of Functionalized Polymers

The functionalization of chitosan is achieved by grafting specific targeting ligands, including folic acid, butyric acid, and phenylalanine, onto the polymer backbone to adjust the nanocarrier properties for biological evaluation. Quantitative assessment in Table 2 demonstrates that this chemical modification is regulated by the initial ligand feeding ratios. Under lower feeding conditions, from 1:20 to 1:100 molar ratios, the actual bound ligand density increases proportionally, maintaining a controlled modification that preserves a fraction of the primary amine groups on the chitosan backbone. For the 1:100 ratio utilized in biological studies, the actual bound ligand reaches its optimal density while leaving a functional fraction of free amine groups available. This primary modification is substantiated by the FTIR profiles presented in Figure 1, confirming the successful amide formation on the chitosan backbone. However, increasing the feeding ratio to 1:300 leads to an over-saturation of the primary amines, rendering the remaining free amines negligible in Table 2.

Under these maximum feeding parameters, the high concentration of the acylating agent promotes a shift in chemical reactivity, initiating esterification at the available hydroxyl sites in addition to the primary amide formation [16]. This dual-functionalization pathway explains the high density of bound ligands recorded at elevated ratios without indicating polymer chain degradation.

### 4.2. Structural Influence on Nanoparticle Formation and Physicochemical Properties

The specific structure of the ligands significantly impacted the formation process during ionic gelation, thus influencing particle size and surface charge. Folic acid (FA), characterized by its rigid aromatic pteridine ring structure, functioned as a bulky, branched scaffold when conjugated to the chitosan backbone. This structural rigidity restricted the rotation and extension of the polymer chains, physically hindering tight packing [17,18] and resulting in the largest average particle size of 263.5 nm. In contrast, butyric acid (BA), a short-chain fatty acid (C4), possesses high flexibility and low steric hindrance. The degree of deacetylation (DDA) and molecular weight (MW) of the chitosan used affect the final nanoparticle parameters. A DDA of 82.5% was utilized to provide primary amine groups (-NH_2_) along the polymer chain, ensuring reactive sites for covalent attachment with FA, PA, and BA ligands while leaving free positive charges to react with TPP during ionic gelation. Low molecular weight chitosan (50–190 kDa) was used because shorter polymer chains lower solution viscosity and reduce chain entanglement, allowing the molecules to pack together during TPP addition. Its conjugation maintained a more linear polymer arrangement, promoting efficient fabrication into compact nanoparticles [19] with the smallest size (128.2 nm). The observed influence of ionic strength and pH on resulting morphology is consistent with other reports on doxorubicin-loaded chitosan nanoparticles [20,21].

Surface charge analysis via zeta potential revealed that electron-withdrawing aromatic groups present in FA and PA decreased the electron density of the nearby amino groups on the chitosan chain, consequently leading to lower surface charges (17.05 mV and 15.59 mV, respectively). In contrast, BA induced no significant electron-withdrawing effect, maintaining a high positive zeta potential. (23.27 mV). This high positive charge is a critical factor, enhancing initial cellular contact and adhesion via robust electrostatic interactions with the negatively charged components of the cell membrane [22,23].

The reported PDI values for CRCSNP (0.162), CRFANP (0.174), CRBANP (0.349), and CRPANP (0.340) demonstrate a physical shift following ligand conjugation. While the unmodified CRCSNP displays a narrow size distribution, ligand conjugation induces steric hindrance on the polymer surface, causing a broader hydrodynamic size distribution across the functionalized configurations. This physical polydispersity presents a technical challenge for batch-to-batch consistency during large-scale clinical translation and regulatory approval. Although the current biological profiles exhibit reproducible antitumor efficacy (Table 5) and stable cellular uptake (Table 6) with narrow standard deviations, further optimization of the fabrication method is required for downstream clinical translation. Future scaling up will utilize advanced manufacturing techniques, such as microfluidic mixing or high-pressure homogenization, to control particle size distribution and minimize PDI variance before clinical evaluation.

### 4.3. Encapsulation Efficiency and Drug Loading Optimization

The evaluation of encapsulation efficiency was based on theoretical calculation and protocols adapted from verified literature, where hydroalcoholic solvent extraction combined with sonication demonstrated high recovery rates (>95%) for encapsulated hydrophobic drugs [20]. Based on this established methodology, the current formulation was strategically designed using a 5% (*w*/*w*) initial curcumin-to-chitosan ratio to maintain an optimal physicochemical equilibrium. Preliminary optimization indicated that while higher initial drug feeding could slightly increase the absolute drug loading (%LC), it induced a sharp reduction in entrapment efficiency (%EE) due to polymer core saturation. By maintaining a 5% initial load, a high %EE (77–80%) was achieved, preventing curcumin waste outside the particles while ensuring a stable formulation. This specific loading level provides sufficient therapeutic efficiency, as evidenced by the significantly lower IC_50_ values in Table 5 and enhanced cellular internalization in Table 6 of the functionalized systems compared to free curcumin. These results confirm that targeted delivery and improved intracellular accumulation eliminate the requirement for a high curcumin content to enhance localized antitumor cytotoxicity. The analysis of encapsulation efficiency was based on protocols adapted from verified literature, where hydroalcoholic solvent extraction combined with sonication demonstrated high recovery rates (>95%) for encapsulated hydrophobic drugs [20].

### 4.4. Mechanisms of Enhanced Cellular Uptake

To ensure the analytical reliability of the intracellular quantitative data, chromatographic evaluation via HPLC requires target analytes to exist in a fully dissolved solution phase. The extraction protocol utilizing 70% methanol with sonication forces a total drug release from the nanocarriers before quantification. This extraction behavior is supported by the in vitro dissolution profiles in Figure 3A, which demonstrate rapid polymer matrix swelling and 70% to 80% curcumin release within 4 h. Considering this high dissolution susceptibility in standard buffer solutions, subsequent exposure to a concentrated organic solvent combined with mechanical sonication breaks down the polymer matrix, releasing the remaining entrapped drug into the liquid medium. This extraction process transforms the internalized formulations into a uniform solution of free curcumin, confirming that the observed uptake differences reflect biological internalization instead of extraction efficiency differences.

The observed cellular accumulation was a synergistic result of physical properties and specific biochemical recognition events. While CRBANP achieved maximum uptake efficiency primarily due to its strong positive charge and smaller size, CRFANP displayed comparable accumulation despite its larger size. This is attributed to folate receptor-mediated endocytosis—a well-established targeting pathway in many cancer cells [24,25]. The specific recognition of the pterin moiety by the overexpressed folate receptors (FRs) facilitates active transport into the cell via clathrin-coated pits. This approach has consistently demonstrated enhanced accumulation compared to passive targeting strategies [26]. For CRPANP, the intermediate uptake suggests that the LAT-1 transporter pathway, although a potential target, may be less efficiently utilized by Caco-2 cells for this specific conjugate compared to the FR pathway [27,28]. Furthermore, previous studies have confirmed that chitosan nanoparticles inherently enhance cellular permeability through non-specific mechanisms such as clathrin-mediated endocytosis [29] and micropinocytosis [30], further contributing to the enhanced delivery observed in this study.

### 4.5. Synergistic Cytotoxicity and pH-Responsive Release

A pivotal finding was that the magnitude of cellular uptake did not exclusively determine the level of cytotoxicity. Despite similar uptake levels in Caco-2 cells, CRBANP was significantly more potent than CRFANP. The enhanced efficacy is caused by the intrinsic histone deacetylase (HDAC) inhibitory activity of butyric acid [30]. The role of butyrate as an HDAC inhibitor is well documented in References [31,32]. By inhibiting HDAC enzymes, BA induces hyperacetylation of histone proteins, leading to chromatin remodeling into an “open” conformation, facilitating the access and expression of pro-apoptotic genes (e.g., BAX, BIM, PUMA) [33], providing a synergistic effect with curcumin’s action on key signaling pathways like NF-κB [34]. This potentiation of chemotherapy by HDAC inhibitors is a known strategy in cancer therapy [35].

The enhanced cytotoxicity of CRBANP is attributed to the nanoparticle-mediated delivery rather than a simple additive effect of the separate components. Free curcumin exhibits poor aqueous solubility and rapid degradation, which limits its cellular internalization in an unencapsulated form. Nanoparticle encapsulation bypasses these solubility limitations through receptor-mediated endocytosis. As confirmed by the FTIR analysis, butyric acid is covalently conjugated to the chitosan backbone, ensuring that the internalization of the nanoparticles drives the co-delivery of both chemical components into the intracellular environment to support the observed synergistic anticancer activity. Although the drug loading is approximately 5% relative to the chitosan mass, this curcumin content is highly sufficient for therapeutic applications. The lower IC_50_ values in Table 5 and the enhanced internalization in Table 6 confirm that this loading configuration delivers an effective intracellular dose, as receptor-mediated targeting and the combined antitumor activity minimize the required curcumin load for selective cytotoxicity.

The biological safety of the functionalized carriers was also evaluated alongside these formulation parameters. Blank chitosan nanoparticles and unconjugated ligands exhibit no significant cytotoxicity at the concentrations utilized in this study, maintaining cell viability above 90% as these baseline components are biocompatible. This confirms that the observed anticancer activity is derived from the encapsulated curcumin and its synergistic interaction with the modified backbone, rather than the intrinsic toxicity of the carriers or free ligands themselves.

The in vitro release data presented in Section 3.3 and Figure 3 confirm the pH-responsive properties of the functionalized nanoparticles. At an acidic pH of 6.5, protonation of the remaining free amino groups of chitosan combined with the partial deprotonation of the butyric acid carboxyl groups (pKa ~ 4.8) induces strong intra- and intermolecular electrostatic repulsion [36,37]. This repulsion drives structural loosening and swelling of the polymer network to accelerate curcumin release, while the neutral environment at pH 7.4 maintains matrix stability and minimizes premature drug leakage. The initial burst release at pH 6.5 rapidly increases localized curcumin concentrations to induce immediate cytotoxicity in cancer cells [34]. This pH-dependent behavior limits drug exposure to healthy tissues at pH 7.4, preventing toxic side effects during circulation [36,37].

The cellular internalization of these functionalized nanoparticles is suggested to involve a combination of passive and active targeting pathways. Initial cellular uptake is partially mediated by non-specific endocytosis, facilitated by the submicron diameter and surface charge detailed in Table 1. However, these physicochemical attributes of nanoparticles are comparable across all formulations; therefore, they cannot explain the distinct variations in cellular accumulation and cytotoxicity observed in Table 3. While these ligand-dependent variations suggest a trend, it is important to note that specific receptor participation remains a preliminary hypothesis. Without definitive competitive inhibition studies, the exact contribution of receptor-mediated pathways cannot be conclusively confirmed. These distinct profiles instead suggest a potential role of ligand-specific interactions that warrant future mechanistic verification.

The variance in the selectivity index (SI) between Caco-2 (46.5) and HT-29 (5.1) cells relates to differential receptor density and HDAC isoform profiles. Baseline expression data confirm that Caco-2 cells possess higher baseline densities of free fatty acid receptors (FFAR2 and FFAR3) than HT-29 cells, promoting increased targeted binding and internalization of the butyric acid formulation [38]. Additionally, HT-29 cells maintain elevated endogenous levels of class I HDACs, particularly HDAC1 and HDAC3, which reduce cell susceptibility to butyric acid-mediated inhibition compared to the response profile in Caco-2 cells [39]. These baseline biological differences cause the lower selectivity index observed in the HT-29 cell line.

### 4.6. Selective Targeting and Safety Profile

The safety of the delivery system was confirmed by the Selectivity Index (SI), which measured the preference for cancer cells over normal HIEC-6 cells. The calculated SI values for all formulations are presented in Table 7. CRBANP achieved the highest SI of 46.5 against Caco-2 cells, showing a higher selectivity profile than the other formulations. The nanoparticle formulations did not reduce normal HIEC-6 cell viability to 50%. As reported in Table 5, the exact IC50 values were not detected within the tested range. Therefore, the maximum tested concentration of 100 µM was used in the SI formula. This calculation results in the greater-than values (>) shown in Table 7. This high selectivity is attributed to the oncogenic overexpression of specific HDAC isoforms (e.g., HDAC 1, 6, and 8) in colorectal adenocarcinoma cells [40,41]. BA induces apoptosis in malignant cells while exhibiting low toxicity toward healthy HIEC-6 cells with normal HDAC levels [42]. In contrast, the lower SI of CRFANP (17.3 for Caco-2) suggests that folate receptors, which are also expressed on some normal tissues, may lead to less specific targeting [43]. This selective toxicity is a highly desirable attribute for targeted cancer therapies. Preliminary screening confirms that a 1% (*v*/*v*) DMSO vehicle control and individual free ligands (butyric acid, folic acid, and phenylalanine) from 5 to 50 µM show no intrinsic cytotoxicity, maintaining high cell viability over 93% across all tested groups.

In summary, the specific structural characteristics of the conjugated ligands directly influenced the physicochemical properties and biological performance of the nanoparticles. The CRBANP formulation provided the best results, as its small size and high positive charge worked together with the anti-cancer activity of butyric acid. By acting as an HDAC inhibitor, the BA-modified system not only delivered the drug effectively but also added a synergistic effect that increased cancer cell death. Most importantly, this system showed a clear preference for Caco-2 cells, meaning it could kill cancer cells while leaving normal cells mostly unharmed. Overall, these results suggest that CRBANP is a strong candidate for further development in targeted colorectal cancer treatment.

### 4.7. Limitations and Future Perspectives

Establishing a baseline understanding of each individual ligand under controlled conditions is necessary before moving toward combination systems. This systematic evaluation isolated the specific effects of FA, PA, and BA modifications within a single controlled system, providing the detailed data needed for future formulation designs. Although this study focused on single-ligand systems without multi-ligand configurations, the synthesis of multi-ligand systems is planned for future investigations to optimize co-conjugation ratios. However, this study is currently limited to in vitro models, meaning that in vivo validation is required to confirm safety and performance. Regarding nanoparticle parameters, the high polydispersity index (PDI) of CRBANP indicates a need to optimize synthesis conditions for better size uniformity. The absence of a control group combining free curcumin and free butyric acid also represents a study limitation, as potential additive effects in the assay solvent were not evaluated. Future investigations must incorporate in vivo models, free drug combination controls, receptor-mediated pathways, and exact extraction recovery rates.

## 5. Conclusions

This study demonstrated that the structural characteristics of conjugated ligands influenced the physical properties and antitumor efficacy of the delivery systems. Among the formulations, CRBANP was the most effective in terms of antitumor potency and selectivity. This formulation displays physical polydispersity, including a higher polydispersity index (PDI 0.349) and a slightly lower encapsulation efficiency (77.24%) compared to the more uniform CRFANP (PDI 0.174, %EE 79.84%). Despite these physical limitations, the high cytotoxicity confirms the therapeutic potential of CRBANP against colorectal cancer cells. Future studies will focus on optimizing fabrication parameters to improve particle uniformity and loading capacity for clinical applications.

## Figures and Tables

**Table 1:** Formulation summary of CS:Ligand conjugates at different molar ratios.

Formulation	CS:Ligand Molar Ratio	Chitosan (mg)	Ligand Amount (mg)		
			FA	BA	PA
FA1	1:20	100	18.0	-	-
FA2	1:50	100	45.0	-	-
FA3	1:100	100	89.0	-	-
FA4	1:200	100	177.0	-	-
FA5	1:300	100	265.0	-	-
BA1	1:20	100	-	3.6	-
BA2	1:50	100	-	8.9	-
BA3	1:100	100	-	17.7	-
BA4	1:200	100	-	35.2	-
BA5	1:300	100	-	53	-
PA1	1:20	100	-	-	6.6
PA2	1:50	100	-	-	16.5
PA3	1:100	100	-	-	33.0
PA4	1:200	100	-	-	66.0
PA5	1:300	100	-	-	99.1

**Table 2:** Summary of CS:Ligand conjugation parameters at various molar ratios.

Parameters	CS: Ligand Molar Ratio				
	1:20	1:50	1:100	1:200	1:300
Conjugation efficiency (%)					
CS:FA	67.53 ± 2.15 ^a^	66.61 ± 1.88 ^a^	80.69 ± 0.65 ^b^	88.87 ± 0.24 ^c^	83.07 ±0.11 ^b^
CS:BA	73.98 ± 2.34 ^a^	70.66 ± 1.15 ^a^	79.94 ± 0.78 ^b^	82.31 ± 0.56 ^c^	84.43 ± 0.20 ^c^
CS:PA	86.55 ± 1.40 ^a^	62.70 ± 1.83 ^b^	69.95 ± 0.27 ^c^	35.22 ± 1.65 ^d^	24.33 ± 0.65 ^e^
Actual Ligand:CS molar ratio (mol/mol)					
CS:FA	42.49 ± 2.76 ^a^	102.27 ±2.52 ^b^	244.25 ± 3.29 ^c^	476.04 ± 3.65 ^d^	668.15 ± 1.72 ^e^
CS:BA	16.70 ± 1.93 ^a^	40.39 ± 2.12 ^b^	92.22 ± 4.50 ^c^	164.47 ± 6.47 ^d^	241.64 ± 3.61 ^e^
CS:PA	19.25 ± 2.56 ^a^	33.26 ± 2.51 ^b^	70.93 ± 1.14 ^c^	71.66 ± 4.44 ^c^	74.11 ± 1.46 ^c^
Amine available (%)					
CS:FA	92.03 ± 0.28 ^a^	79.69 ± 0.16 ^b^	58.94 ± 0.24 ^c^	27.34 ± 0.30 ^d^	Negligible
CS:BA	92.37 ± 0.65 ^a^	79.81 ± 0.69 ^b^	58.20 ± 1.63 ^c^	27.59 ± 2.33 ^d^	Negligible
CS:PA	92.48 ± 0.87 ^a^	81.28 ± 0.83 ^b^	63.24 ± 0.51 ^c^	26.30 ± 1.27 ^d^	Negligible

Values with different superscript letters (^a–e^) within the same row indicate statistically significant differences (*p* < 0.05) between the ligand-functionalized formulations. Values are mean ± SD (*n* = 3).

**Table 3:** Physicochemical characterization of various ligand-conjugated chitosan nanoparticles.

Formulation	Size (nm)	PDI	Zeta Potential (mV)	%EE	%LE
CRCSNP	162.9 ± 1.2	0.162 ± 0.009	10.34 ± 0.95	78.64 ± 1.95	4.92 ± 0.55
CRFANP	263.5 ± 8.4	0.174 ± 0.037	17.05 ± 0.74	79.84 ± 1.75	5.53 ± 0.59
CRBANP	128.2 ± 3.0	0.349 ± 0.005	23.27 ± 1.10	77.24 ± 8.73	4.89 ± 0.22
CRPANP	138.0 ± 2.9	0.340 ± 0.028	15.59 ± 2.14	79.77 ± 0.55	4.87 ± 0.03

Data were presented as triplicate (*n* = 3) and mean ± SD.

**Table 4:** Physicochemical characteristics of chitosan nanoparticles conjugated with various ligands, as determined by DLS and NTA techniques.

Parameters	Ligand Type			
	Unmodified CS	Folic Acid	Butyric Acid	Phenylalanine
DLS—Size (nm)	162.9 ± 1.2	263.5 ± 8.4	128.2 ± 3.0	138.0 ± 2.9
NTA—Size (nm)	213	268	304	263
Mode [nm]	127.5	107.5	232.5	107.5
D10 [nm]	78	90	98	92
D50 [nm]	158	201	255	198
D90 [nm]	429	529	567	512
Concentration [particles/mL]	1.91 × 10^12^	1.85 × 10^12^	1.69 × 10^12^	1.93 × 10^12^

Note: CS: Chitosan; FA: Folic acid; BA: Butyric acid; PA: Phenylalanine; DLS: Dynamic Light Scattering; NTA: Nanoparticle Tracking Analysis.

**Table 5:** Comparative IC_50_ values of free curcumin and functionalized chitosan nanoparticles in Caco-2 and HT-29 cells (24 h).

Group	IC_50_ (µM)		
	Caco-2	HT-29	HIEC-6
Free Curcumin	20.97 ± 4.12 ^a^	42.47 ± 1.53 ^a^	60.45 ± 2.84
CRCSNP	7.54 ± 1.25 ^b^	23.21 ± 2.24 ^b^	>100
CRPANP	7.40 ± 2.66 ^b^	24.96 ± 9.89 ^b^	>100
CRFANP	3.49 ± 0.73 ^c^	11.55 ± 1.70 ^c^	>100
CRBANP	1.30 ± 0.43 ^d^	11.90 ± 1.65 ^c^	>100

IC_50_ values designated as >100 µM indicate that 50% inhibition of cell viability was not achieved at the maximum concentration tested. Data are presented as mean ± standard deviation (SD), *n* = 6. ^a–d^ Different superscript letters within each cell line column indicate significant differences among the formulations (*p* < 0.05).

**Table 6:** Quantitative cellular uptake of curcumin (nM/mg protein) by Caco-2 and HT-29 cells after incubation for 2 h with different formulations.

	Cellular Uptake (nM/mg Protein)	
	Caco-2	HT-29
Free Curcumin	8.11 ± 2.59 ^a^	5.16 ± 2.33 ^a^
CRCSNP	12.06 ± 1.56 ^b^	4.97 ± 1.73 ^a^
CRFANP	20.97 ± 3.12 ^d^	19.64 ± 3.02 ^c^
CRPANP	15.40 ± 0.68 ^c^	12.88 ± 2.24 ^b^
CRBANP	21.92 ± 2.11 ^d^	22.09 ± 2.73 ^c^

Data are presented as mean ± standard deviation (SD), *n* = 3. Different superscript letters within the same column indicate significant differences between formulations (*p* < 0.05).

**Table 7:** Selectivity Index (SI) values of various curcumin formulations against colorectal cancer cells (Caco-2 and HT-29) compared with normal HIEC-6 cells.

Formulations	Selectivity Index: SI	
	Caco-2	HT-29
Free curcumin	2.9	1.4
CRCSNP	>8.0	>2.6
CRFANP	>17.3	>5.2
CRPANP	>8.2	>2.4
CRBANP	>46.5	>5.1

Selectivity Index (SI) was calculated using the maximum tested concentration (100 µM) as a conservative IC_50_ value for HIEC-6 cells. Thus, these SI values are presented as greater-than values (>).

## Data Availability

The original contributions presented in this study are included in the article. Further inquiries can be directed to the corresponding author.

## Abbreviations

The following abbreviations are used in this manuscript:

CS	Chitosan
FA	Folic acid
BA	Butyric acid
PA	Phenylalanine
CRCSNP	Curcumin loaded chitosan nanoparticles
CRFANP	Curcumin loaded folic acid conjugated chitosan nanoparticles
CRBANP	Curcumin loaded butyric acid conjugated chitosan nanoparticles
CRPANP	Curcumin loaded phenylalanine conjugated chitosan nanoparticles
DCC	N,N′-dicyclohexylcarbodiimide
DMAP	4-dimethylaminopyridine
DCU	dicyclohexylurea
